# Important role of N108 residue in binding of bovine foamy virus transactivator Tas to viral promoters

**DOI:** 10.1186/s12985-016-0579-2

**Published:** 2016-06-30

**Authors:** Tiejun Bing, Suzhen Zhang, Xiaojuan Liu, Zhibin Liang, Peng Shao, Song Zhang, Wentao Qiao, Juan Tan

**Affiliations:** Key Laboratory of Molecular Microbiology and Technology, Ministry of Education, College of Life Sciences, Nankai University, Tianjin, 300071 China

**Keywords:** Bovine foamy virus, Infectious clone, BTas, Promoter

## Abstract

**Background:**

Bovine foamy virus (BFV) encodes the transactivator BTas, which enhances viral gene transcription by binding to the long terminal repeat promoter and the internal promoter. In this study, we investigated the different replication capacities of two similar BFV full-length DNA clones, pBS-BFV-Y and pBS-BFV-B.

**Results:**

Here, functional analysis of several chimeric clones revealed a major role for the C-terminal region of the viral genome in causing this difference. Furthermore, BTas-B, which is located in this C-terminal region, exhibited a 20-fold higher transactivation activity than BTas-Y. Sequence alignment showed that these two sequences differ only at amino acid 108, with BTas-B containing N108 and BTas-Y containing D108 at this position. Results of mutagenesis studies demonstrated that residue N108 is important for BTas binding to viral promoters. In addition, the N108D mutation in pBS-BFV-B reduced the viral replication capacity by about 1.5-fold.

**Conclusions:**

Our results suggest that residue N108 is important for BTas binding to BFV promoters and has a major role in BFV replication. These findings not only advances our understanding of the transactivation mechanism of BTas, but they also highlight the importance of certain sequence polymorphisms in modulating the replication capacity of isolated BFV clones.

## Background

Foamy viruses (FVs) form the only genus in the *Spumaretrovirinae* subfamily of the *Retroviridae* family and are widespread in various animals, including simian [1, 2], bovine [3], equine [4] and feline [5] species. FVs have extensive cellular tropism and can infect many different kinds of cells resulting in lytic replication (Cf2Th cells and BHK-21 cells) or persistence (HEK293T cells and HeLa cells). However, FVs are nonpathogenic in their natural hosts or in experimentally infected animals. The genomic nature, replication strategy, and gene expression of FVs are different in many aspects from other retroviruses. The replication of FVs genes is dependent on two distinct promoters: the 5’ long terminal repeat (LTR) regulates the expression of three structural genes (*gag*, *pol* and *env*), and an internal promoter (IP) located between the *env* gene and the 3’ LTR also regulates the expression of two regulatory genes (*tas* and *bet*). Tas, a transcriptional transactivator, is essential for FV replication. The Tas protein binds directly to a DNA sequence in the viral LTR promoter and IP to enhance viral gene transcription [6–10]. Nuclear localization of the prototype FV (PFV) Tas is essential for its transactivation activity and is regulated by Tas acetylation [11, 12].

Unlike PFV that transmit through both cell-to-cell and cell-free ways, bovine foamy virus (BFV) infection is tightly cell-associated. BFV Tas (BTas) contains at least two functional domains, including the N-terminal DNA-binding domain (residues 1 to 133) and the C-terminal activation domain (residues 198 to 249) [10, 13]. BTas has no classical nuclear localization signal (NLS), yet it accumulates mainly in the nucleus [14, 15]. Furthermore, BTas stimulates viral gene transcription through binding to a region at nucleotide positions −380 to −140 in the BFV LTR or to a region at nucleotide positions 9205 to 9276 in the IP [10]. Besides, BTas acetylation and dimerization are required for its transactivation function and are crucial for the replication of BFV [15, 16].

We previously generated the full-length BFV infectious clone pBS-BFV-Y in 2003 that could produce infectious BFV and induce cytopathic effects (CPE) in primary fetal bovine lung (FBL) cells [17]. We subsequently isolated 18 infectious BFV clones including pBS-BFV-B in 2011 and found that the pBS-BFV-Y and pBS-BFV-B viruses had markedly different replication abilities. In this study, we identified the BTas protein as one of the determinants of this difference and further showed that a single amino acid at position 108 in this protein could greatly modulate BTas transactivation through regulating BTas binding to the LTR or the IP.

## Methods

### Reagents

4', 6-Diamidino-2-phenylindole (DAPI) and mouse anti-tubulin antibody were purchased from Sigma (St. Louis, MO). Antibodies against the Myc tag and Flag tag were obtained from Santa Cruz Biotechnology (Dallas, USA). Fluorescein-conjugated anti-rabbit secondary antibodies were obtained from Jackson ImmunoResearch Laboratories (West Grove, PA). Mouse anti-Gag [18] and anti-BBet [19] and rabbit anti-BTas [16] were generated after immunization with full-length BFV3026 Gag, BBet and BTas proteins, respectively, purified from bacteria. These polyclonal antisera were used for immunofluorescence assays and Western blotting.

### Plasmids

BFV3026 full-length genomic DNA clones, pBS-BFV-Y (unpublished) and pBS-BFV-B [17], were generated by our lab. pLTR-Luc and pIP-Luc were constructed as described previously [15, 20]. pCMV-AD-BTas (1–133 aa) was cloned in the pCMV-AD (Stratagene, La Jolla, USA) vector. pCMV-BTas-Y and pCMV-BTas-B vectors were constructed by exchanging individual PCR fragments in the pcDNA3.1(+) vector. pBFV-3'LTR-B-Luc, pBFV-3'LTR-Y-Luc, pBFV-5'LTR-B-Luc and pBFV-5'LTR-Y-Luc were constructed by inserting corresponding LTR fragments into the pGL3basic (Promega) vector. Glutathione S-transferase (GST)-BTas-Y, GST-BTas-B and GST-BTasD108S were generated by inserting different *btas* genes into the pGEX6P-1 DNA vector. All new constructs were confirmed by sequencing.

### Cells and viruses

HEK293T, HeLa, Cf2Th, BHK-21 and BFVL (BHK-21-derived indicator cells containing a *luciferase* gene under the control of the BFV LTR) [21] cells were maintained in Dulbecco’s modified Eagle’s medium (high glucose) supplemented with 10 % fetal bovine serum (Hyclone, Victoria, Australia), 50 IU/mL penicillin and 50 μg/mL streptomycin. Cells were maintained in a humidified atmosphere containing 5 % CO_2_ at 37 °C. HeLa and HEK293T were transfected by using PEI (Polysciences, Warrington, USA) or Lipofectamine 2000 (Invitrogen, Carlsbad, USA) in accordance with the manufacturer’s instructions. BFV3026 was isolated from peripheral lymphoid cells by our lab in 1997 [3].

### Hirt DNA extraction

Cf2Th cells (5 × 10^6^) infected with BFV3026 for 2 days were harvested, washed with 10 mL buffer K (20 mM HEPES, 140 mM KCl, 5 mM MgCl2, 1 mM DL-Dithiothreitol [DTT]), and lysed with 250 μL buffer K and 7.5 mL 0.5 % Triton X-100 for 10 min at room temperature. After centrifugation, the pellet was dissolved in 400 μL TE and 90 mL 5 M NaCl at 4 °C overnight. The supernatant after centrifugation was extracted with phenol, chloroform:isoamylalcohol (24:1), and then precipitated for DNA in ethanol with 0.3 M NaAc at −20 °C for 1 h. The DNA pellet was then washed with 70 % ethanol, dissolved in 20 μL TE, and stored at −20 °C.

### Western blotting

Cell lysates were separated by 10 % SDS-PAGE (polyacryl-amide gel electrophoresis). Proteins were transferred onto a polyvinylidene difluoride (PVDF) membrane (Millipore, Billerica, USA). Following incubation in phosphate-buffered saline (PBS) containing 5 % non-fat milk for 45 min at room temperature, the membranes were probed with the indicated primary antibodies for 90 min at room temperature or overnight at 4 °C. After incubation with either goat anti-rabbit or goat anti-mouse secondary antibody, the membranes were treated with enhanced chemiluminescence reagents (Millipore, Billerica, USA), and signals were detected by exposure to X-ray films.

### Luciferase reporter assay

HEK293T cells were seeded at a concentration of 2.5 × 10^5^ cells/well. The following day, cells were transfected with pBS-BFV-B, pBS-BFV-Y or empty vector pBS along with the luciferase plasmid. pCMV-β-gal was co-transfected as a control for transfection efficiency. Forty-eight hours after transfection, cells were harvested in lysis buffer, and luciferase assays were performed using the luciferase reporter assay system (Promega, Madison, USA). The relative luciferase activity was calculated by normalizing the firefly luciferase activity to the β-galactosidase (β-Gal) activity. Three independent transfection experiments were performed.

### Immunofluorescence assay (IFA)

HeLa cells (3 × 10^4^) were seeded on cover slips in 12-well plates for 24 h and then transfected with pcDNA3.1(+), pcDNA3.1(+)-BTas-Y or pcDNA3.1(+)-BTas-B. Forty-eight hours later, cells were washed with PBS twice, followed by fixation in 4 % formaldehyde and permeabilization in 1 % Triton X-100. Indirect immunofluorescence was carried out with BTas antiserum (1:500 dilution) and a FITC-conjugated goat anti-rabbit secondary antibody (1:500 dilution) diluted in antibody solution (3 % BSA, 1 % Triton X-100 and 0.05 % NaN_3_ in PBS). Nuclei of cells were stained with DAPI (0.2 μg/mL). Fluorescence was observed with an Olympus fluorescence microscope.

### Crosslinking assay

HEK293T cells were transfected with pcDNA3.1(+)-BTas-Y, pcDNA3.1(+)-BTas-Y or pcDNA3.1(+). Forty-eight hours later, cells were washed with ice-cold PBS and lysed in cross-linking lysis buffer (20 mM Tris-Cl, pH 7.5, 150 mM EDTA, 0.5 mM Na_3_VO_4_ and 1 % NP-40) supplemented with protease inhibitors. The lysates were sonicated briefly and centrifuged at 15,000 × *g* for 10 min at 4 °C. The supernatant was mixed with cross-linking buffer (0.01 % glutaralde-hyde in 20 mM triethanolamine, pH 8.2 and 10 mM NaCl) and incubated at room temperature for 30 min. The cross-linking reaction was stopped by addition of SDS-PAGE loading buffer, and proteins were separated by 12 % SDS-PAGE, followed by immunoblotting with an anti-BTas antibody.

### Electrophoretic mobility shift assay (EMSA)

GST fusion proteins were expressed in *Escherichia coli* strain BL21 and subsequently purified by glutathione sepharose 4B beads (GE Healthcare, Uppsala, Sweden). Protein concentrations were determined by the Bradford assay. The binding reaction was carried out with 100 ng purified protein. The BFV LTR probe (613–635) and IP probe (9243–9264) were synthesized and annealed. All probes were labeled by digitonin using the DIG Gel Shift Kit (Roche, Indianapolis, USA). For competition experiments, the following unlabeled competitor oligodeoxynucleotides were added in 20-fold molar excess at the pre-incubation period: BFV LTR, TRE sense: 5′-ATAGCTATTTTAGTAAGTTAGC-3′; BFV IP, TRE sense: 5′-AGAGCTTAAAAATCAA GGTAAC-3′.

### Co-culture assay

BHK-21 cells (1.5 × 10^5^) were seeded in 12-well plates. Twenty-four hours later, the cells were transfected with 1 μg pBS-BFV DNA. The transfected cells were harvested 48 h post-transfection and then co-cultured with BFVL reporter cells (8 × 10^4^). After 48 h of co-cultivation, luciferase activity was measured and normalized to the value of luciferase activity in the uninfected cells.

## Results

### Two BFV full-length DNA clones produce viruses with different replication capacities

**Fig. 1:**
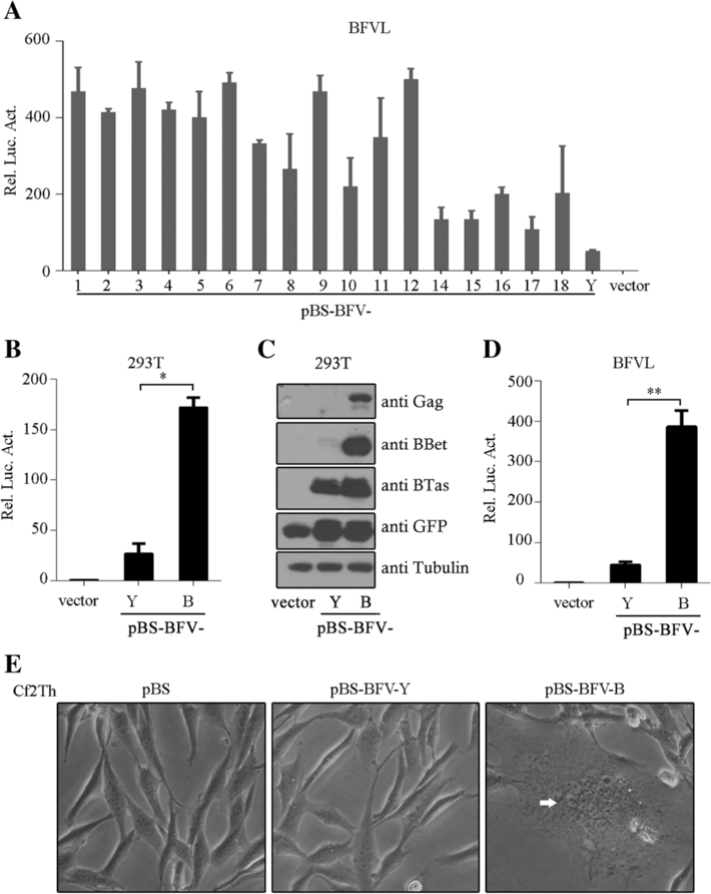
Replication capacities of pBS-BFV-B and pBS-BFV-Y. **a** BFVL cells (8 × 10^4^) were seeded in 12-well plates. After 24 h, 1 μg pBS-BFV DNA together with 50 ng pCMV-β-gal were transfected into BFVL cells, and luciferase activity was measured 48 h later. Relative luciferase activities (Rel. Luc. Act.) are shown. **b** HEK293T cells (2.5 × 10^5^) were transfected with 1 μg pBS-BFV-B, pBS-BFV-Y or empty vector pBS together with reporter plasmids, 20 ng pBFV-LTR-Luc and 10 ng pCMV-β-gal. Forty-eight hours later, the cells were harvested and analyzed using a luciferase activity assay. **c** Cell lysates from **b** were harvested for Western blotting using indicated antibodies. **d** BFVL cells (8 × 10^4^) were transfected with 1 μg pBS-BFV-B, pBS-BFV-Y or empty vector pBS together with 50 ng pCMV-β-gal. Forty-eight hours later, the cells were harvested and analyzed using a luciferase activity assay. **e** Cf2Th cells (1.5 × 10^5^) were transfected with 2 μg pBS, pBS-BFV-Y or pBS-BFV-B. Seventy-two hours later, syncytium was detected in the pBS-BFV-B-transfected Cf2Th cells (*white arrows*). Luciferase activities shown are averages from three independent experiments. Error bars indicate standard deviations (SD). *P < 0.05 (unpaired *t* test), **P < 0.01 (unpaired *t* test)

In order to characterize BFV3026 at the molecular level, we.amplified the full-length genomic DNA of BFV3026 from the Hirt DNA extracted from infected Cf2Th cells that can support lytic BFV infection. The amplified virus DNA fragments were inserted into the pBluescript SK- (pBS) vector to generate infectious clones [17]. We generated 18 such clones in 2011 and tested their replication abilities by transfection into BFVL indicator cells expressing firefly luciferase under the control of the BFV promoter. As shown in Fig. 1a, all of the clones were able to activate the expression of luciferase driven by the BFV promoter. Compared to the pBS-BFV-Y clone that was generated in 2003 by amplifying viral DNA extracted from BFV-infected primary FBL cells, these new clones all exhibited higher levels of transactivation activity. In order to investigate the genetic basis of this enhancement, one of the highly active clones #12, hereafter referred to as pBS-BFV-B, was further investigated.

As shown in Fig. 1b, the pBS-BFV-B DNA clone transactivated the BFV LTR ~8-fold more potently than did the pBS-BFV-Y clone. When viral accessary proteins BTas and BBet as well as structural protein Gag were examined by Western blotting, higher levels of these viral proteins were observed for pBS-BFV-B than pBS-BFV-Y in HEK293T cells and BFVL cells (Fig. 1c and d). Lastly, these two infectious clones were transfected into Cf2Th cells, and formation of syncytium was monitored daily under a bright-field microscope. Three days post-infection, typical CPE were observed in cells transfected with pBS-BFV-B but not with empty vector or pBS-BFV-Y (Fig. 1e). All together, these data demonstrate that pBS-BFV-B replicated to a greater level compared to pBS-BFV-Y.

### C-terminal region of BFV determines different replication capacities of pBS-BFV-Y and pBS-BFV-B

**Fig. 2:**
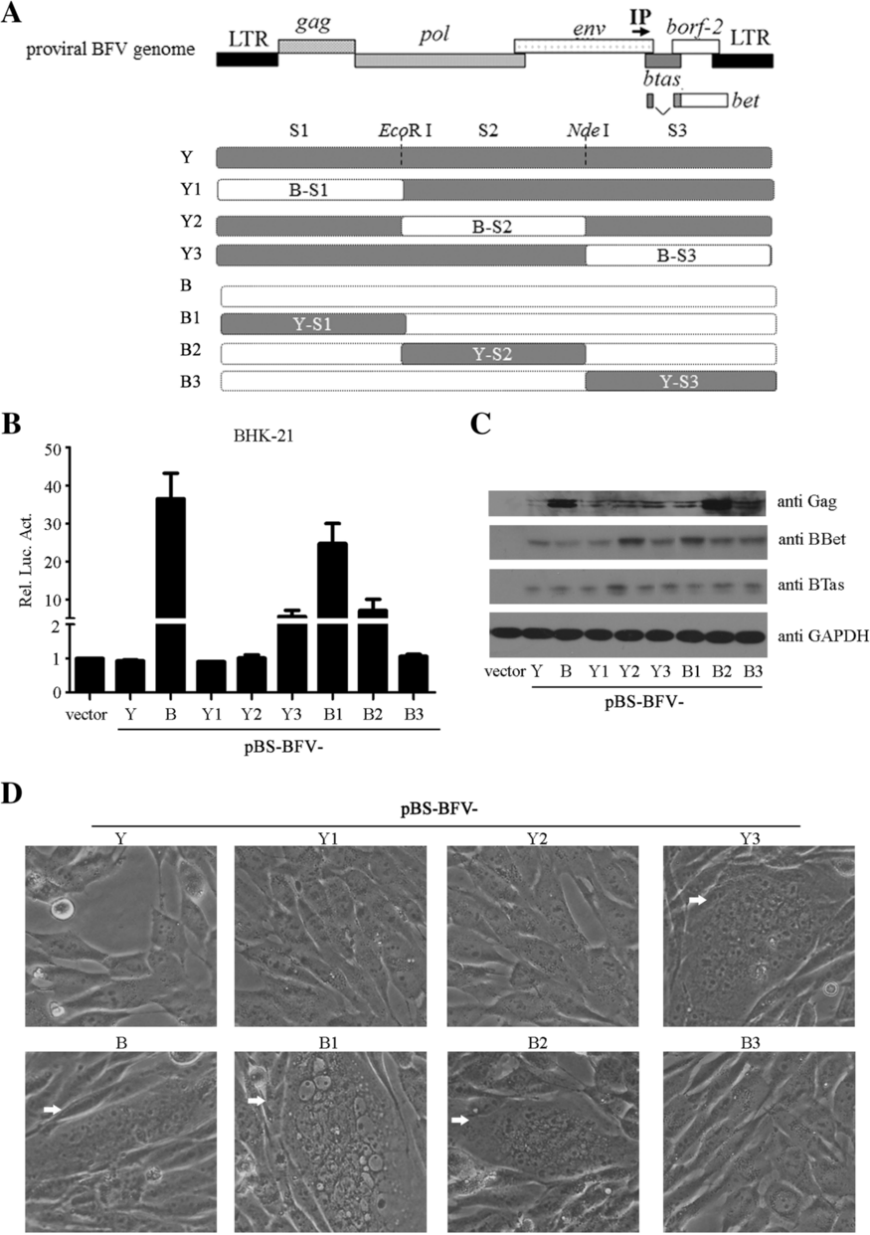
Activities of chimeric pBS-BFV clones. **a** The BFV3026 proviral genome is shown at the top. The gray box depicts the BFV3026 proviral genomic DNA of pBS-BFV-Y, and the white box represents that of pBS-BFV-B. The full-length viral DNA was divided into three segments, S1, S2 and S3, based on the single restriction enzyme sites of *Eco*R I and *Nde* I. The new chimeric clones were generated by exchanging the fragments between pBS-BFV-Y and pBS-BFV-B plasmids. **b**-**c** BHK-21 cells (1.2 × 10^5^) were transfected with 1 μg pBS, pBS-BFV-B, pBS-BFV-Y or different chimeric mutants as indicated. At 48 h post-transfection, cells were harvested and co-cultured with BFVL cells **b**. Transfected BHK-21 cells were also analyzed by Western blotting to measure viral protein expression **c**. **d** Cf2Th cells (1.5 × 10^5^) were transfected with 2.5 μg pBS, pBS-BFV-B, pBS-BFV-Y or different chimeric mutants as indicated. Seventy-two hours later, syncytia (*white arrows*) were recorded using a microscope

In order to determine which part of the viral genome is responsible for the higher replication capacity of pBS-BFV-B, we divided the viral genome into three segments using the *Eco*R I and *Nde* I restriction enzyme sites, designated as S1, S2 and S3. Six chimeric clones were generated as shown in Fig. 2a. To evaluate the replication abilities of these chimeric clones, they were transfected into BHK-21 cells that support persistent BFV replication, followed by co-cultivation with BFVL cells. When the S3 fragment in pBS-BFV-Y was replaced by S3 from pBS-BFV-B, the chimeric clone Y3 thus generated showed a higher replication ability than Y, Y1 or Y2 (Fig. 2b). Results of the pBS-BFV-B mutants showed that substitution of Y-S3 for the counterpart sequence severely diminished the replication ability of B clone (Fig. 2b). Levels of the replication capacities of these BFV clones correlated with levels of viral protein expression (Fig. 2c) and the number of syncytia formed in transfected Cf2Th cells (Fig. 2d). Notably, the Y3 clone induced more CPE than Y clone, and the B3 clone lost the ability to generate CPE. Together, these results suggest that the C-terminal region of the BFV genome (S3 fragment) plays an important role in viral replication.

### Residue N108 is important for the transactivation activity of BTas

**Fig. 3:**
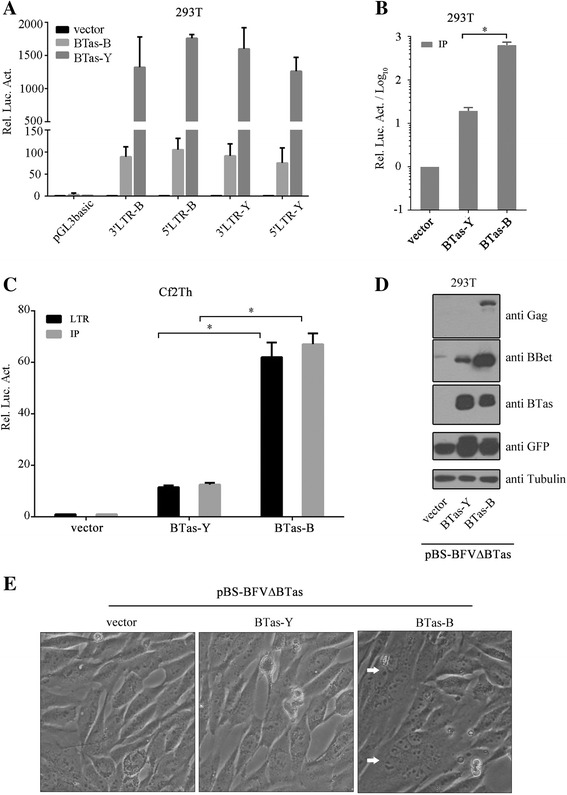
BTas transactivation of LTR and IP. **a** HEK293T cells (2.5 × 10^5^) were transfected with 20 ng pGL3basic, BFV-3'LTR-B-Luc, pBFV-3'LTR-Y-Luc, pBFV-5'LTR-B-Luc or pBFV-5'LTR-Y-Luc together with 100 ng pCDNA3.1+, BTas-B or BTas-Y plasmid as indicated. A luciferase activity assay was performed 48 h post-transfection. **b** HEK293T cells (2.5 × 10^5^) were transfected with 100 ng BTas, BTas-B or BTas-Y eukaryotic expression plasmid together with 20 ng pBFV-IP-Luc. Luciferase activity was measured 48 h post-transfection. **c** Cf2Th cells (8 × 10^4^) were transfected with 200 ng vector, BTas or BTas-B eukaryotic expression plasmid together with 50 ng pBFV-LTR-Luc or pBFV-IP-Luc. Forty-eight hours later, cells were harvested and analyzed using a luciferase activity assay. All results shown are averages from three independent experiments. Error bars indicate SD. *P < 0.05 (unpaired *t* test). **d** HEK293T cells (2.5 × 10^5^) were transfected with 200 ng empty vector, BTas-B or BTas-Y eukaryotic expression plasmid together with 800 ng pBS-BFV∆BTas and 50 ng pEGFP-C3. Forty-eight hours later, cells were harvested for Western blotting using indicated antibodies. **e** Cf2Th cells (1.5 × 10^5^) were transfected with 200 ng empty vector, BTas-B or BTas-Y eukaryotic expression plasmid together with 800 ng pBS-BFV∆BTas. Seventy-two hours later, syncytium was detected in pBS-BFV-B-transfected Cf2Th cells (*white arrows*)

The S3 segment contains the C-terminal region of *env*, *btas*, *bbet* and 3' LTR (Fig. 2a). In order to further identify the viral gene responsible for the difference in replication capacity between pBS-BFV-Y and pBS-BFV-B, we sequenced their S3 DNA fragments. The sequencing results revealed amino acid changes in the Env, BTas and BBet proteins (Table 1). We first examined how the changes affected the transactivation ability of BTas. The reporter plasmids containing LTRs were transfected together with the pcDNA3.1(+) vector or BTas expression plasmids. As shown in Fig. 3a, the basal activity of each LTR was as low as that of the empty vector pGL3basic in the absence of BTas, and different BFV LTRs responded to the transactivation of BTas of different origins at similar levels. Strikingly, BTas-B activated BFV LTR promoters by almost 1500-fold, which is ~10-fold higher than did BTas-Y. This potent activity of BTas-B was also observed with the IP (Fig. 3b) and in Cf2Th cells (Fig. 3c). In addition, when the transactivation ability of BTas was examined by co-transfection with a BTas-deficient infectious clone [19], expression levels of Gag and BBet were found to be higher in cells co-transfected with BTas-B than with BTas-Y (Fig. 3d). In support of its greater transactivation capacity, BTas-B, but not BTas-Y, helped the BTas-deficient clone to induce CPE in Cf2Th cells three days post-transfection (Fig. 3e).

**Table 1:** Sequences of S3 region from BFV-Y and BFV-B

Gene		Nuclear acids			Amino acids	
	Position	pBS-BFV-Y	pBS-BFV-B	Position	pBS-BFV-Y	pBS-BFV-B
*env*	7965	C	G	447	L	V
	8278	C	A	551	A	D
	8373	A	G	583	T	A
	8547	G	A	641	A	T
	8907	G	A	761	V	I
*btas*	9884	G	A	108	D	N
*bbet*	10083	G	A	72	V	I

**Fig. 4:**
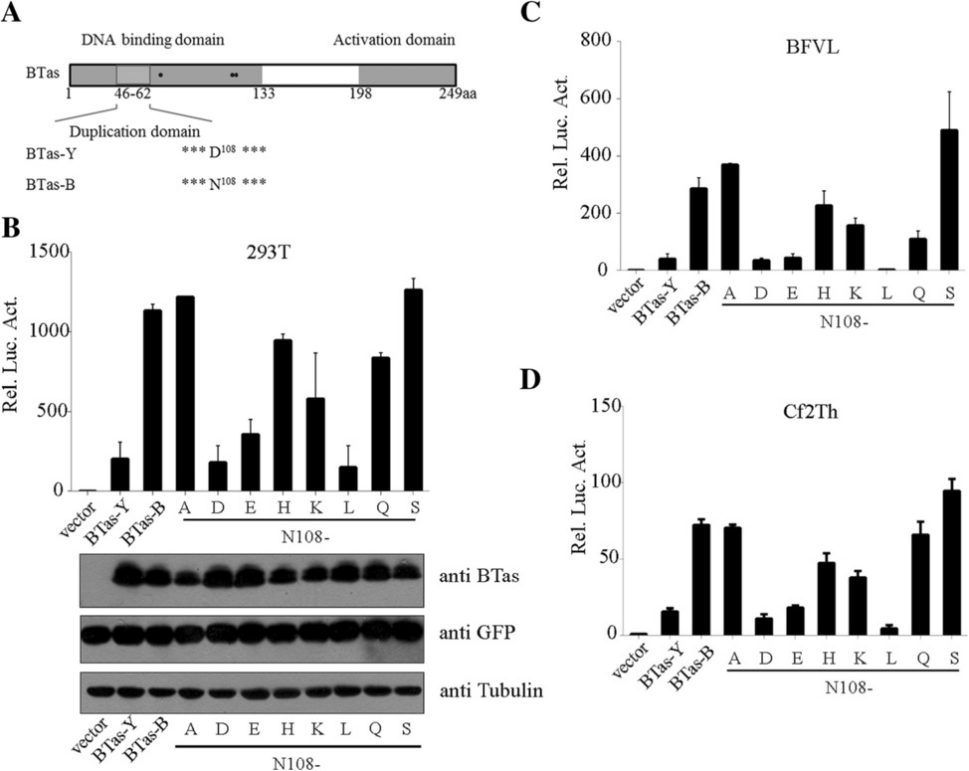
Residue N108 is important for BTas transactivation. **a** Schematic representation of the BTas protein. BTas DNA binding domain (1–133 aa) and activation domain (198–249 aa) on the top are shown in the *grey box*. The dimerization domain (46–62 aa) is marked with a *lighter grey box*. Solid dots represent three lysines at positions 66, 109 and 110 which are also acetylated. Amino acids at position 108 are shown for BTas-Y and BTas-B. **b** HEK293T cells were transfected with BTas-Y, BTas-B, mutated BTas-B plasmids or empty vector (100 ng) together with 20 ng pBFV-LTR-Luc, 50 ng pEGFP-C3 and 10 ng pCMV-β-gal. Luciferase activity was measured 48 h post-transfection. Levels of BTas, GFP and tubulin proteins were measured by Western blotting. **c**-**d** pcDNA3.1(+), BTas-Y, BTas-B and BTas-B mutants were expressed in BFVL **c** and Cf2Th cells **d**. Luciferase activity was determined at 48 h post-transfection

BTas has the DNA binding domain (1–133 aa), activation domain (198–249 aa), dimerization domain (46–62 aa) and three lysines at positions 66, 109 and 110 that are acetylated by p300 (Fig. 4a). The sequencing analysis showed that BTas-Y and BTas-B differed only in the amino acid at position 108 with D108 for BTas-Y and N108 for BTas-B (Fig. 4a). To characterize the function of residue N108, we mutated it to different amino acids (Fig. 4b). The transactivation ability of BTas-B was decreased to the level of that of BTas-Y when N108 was mutated to aspartic acid (D). The transactivation ability was also reduced when N108 was mutated to glutamic acid (E) or leucine (L). No significant difference was observed when N108 was mutated to alanine (A), histidine (H) or serine (S), with BTas-B-N108S even showing a slightly higher transactivation activity (Fig. 4b). Similar results were obtained when experiments were performed in BFVL or Cf2Th cells (Fig. 4c and d). Together, these findings demonstrate the importance of residue N108 in BTas transactivation.

### Residue N108 does not affect BTas subcellular localization or dimerization

**Fig. 5:**
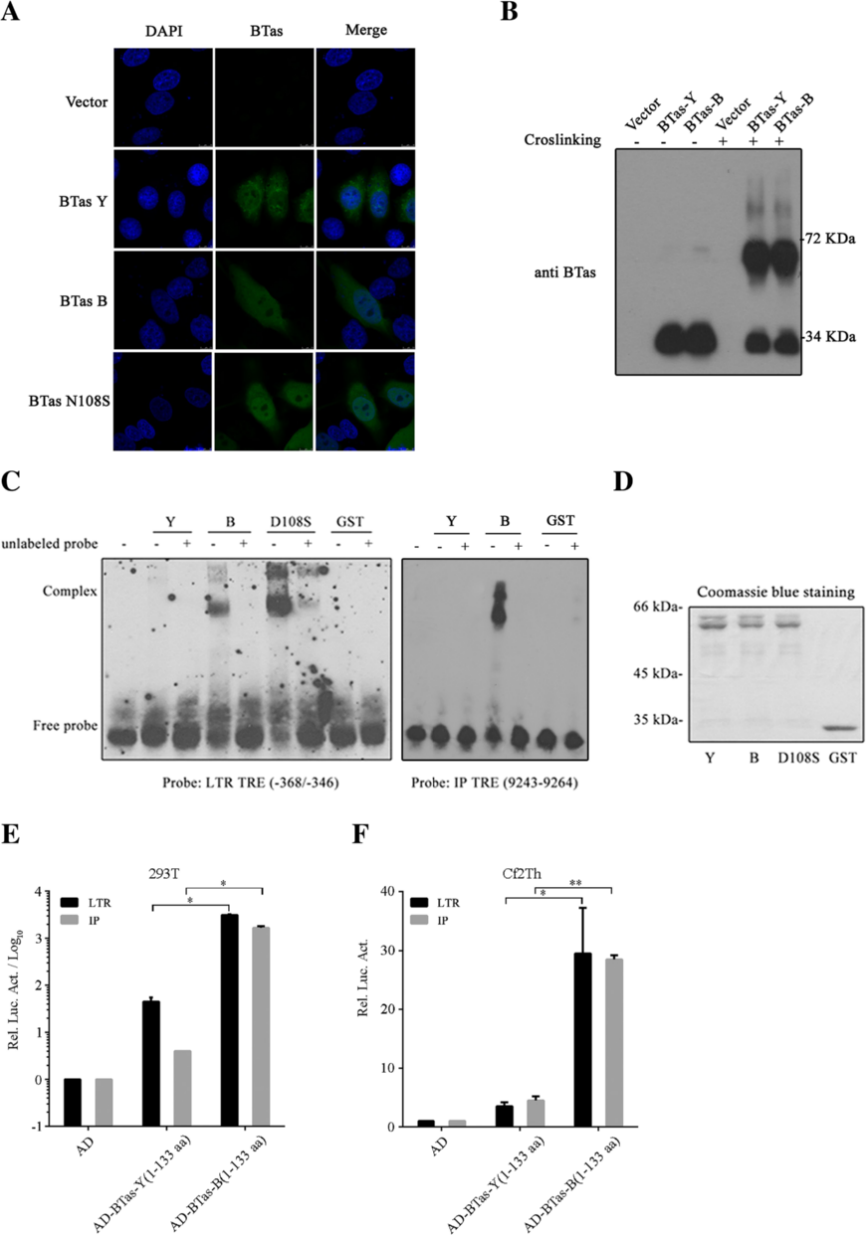
Subcellular localization, dimerization and DNA binding of BTas proteins. **a** HeLa cells (5 × 10^4^) were transfected with pcDNA3.1(+), BTas-Y, BTas-B or BTas-B-N108S. Indirect immunofluorescence was performed 48 h post-transfection to visualize subcellular localization of different BTas proteins (with rabbit anti-BTas antibody at 1:400 dilution and FITC-conjugated goat anti-rabbit secondary antibody at 1:500 dilution). Nuclei were stained with DAPI (0.2 μg/mL). **b** HEK293T cells were transfected with BTas-Y, BTas-B or empty vector. Forty-eight hours later, cell lysates were incubated with or without the cross-linking buffer followed by Western blotting. Positions of BTas monomers (M) and dimers (D) are indicated. **c** EMSAs were performed with purified GST-BTas-B, GST-BTas-Y and GST-BTas-B-N108S and GST proteins. DNA probes from BFV-LTR TRE (−368/-346) or BFV-IP TRE (9243–9264) were labeled with digitonin. **d** Recombinant proteins GST-BTas-B, GST-BTas-Y, GST-BTas-B-N108S and GST used in EMSA were detected by Coomassie blue staining. (**e**) DNA binding ability of BTas was analyzed using a mammalian expression system in HEK293T cells. HEK293T cells (2.5 × 10^5^) were transfected with 100 ng empty vector, pCMV-AD-BTas-B (1–133 aa) or pCMV-AD-BTas-Y (1–133 aa) together with 20 ng pBFV-LTR-Luc or pBFV-IP-Luc. Forty-eight hours later, luciferase activities were measured as described in Materials and Methods. **f** Experiments in **e** were carried out in Cf2Th cells. All results shown are the average from three independent experiments. Error bars indicate SD. *P < 0.05 (unpaired *t* test), **P < 0.01 (unpaired *t* test)

Since BTas exerts its transactivation ability in the nucleus, we tested whether N108 has a role in the cellular distribution of BTas. To this end, plasmids expressing various BTas proteins were transfected into Hela cells, and its subcellular localization was evaluated by confocal microscopy. The results showed that all BTas proteins were distributed both in the nucleus and the cytoplasm. BTas-Y showed granular structures in the cytoplasm, whereas BTas-B and BTas-B-N108S proteins were largely dispersed in the cytoplasm (Fig. 5a).

Given the critical role of the dimerization domain in the transactivation function of BTas [15], we also examined whether residue N108 affects BTas dimerization by performing a cross-linking assay. The results showed that both BTas-Y and BTas-B formed dimers after crosslinking (Fig. 5b), indicating that residue N108 does not affect dimerization of the BTas protein.

### Residue N108 is important for BTas binding to LTR and IP

The DNA binding domain of BTas binds to viral LTR or IP and is essential for BTas to transactivate viral gene expression [10]. We therefore hypothesized that the amino acid at position 108 may be involved in the interaction of BTas with DNA. To test this hypothesis, EMSA was carried out to evaluate the binding of BTas to BFV LTR and IP probes as described previously [10]. First, GST-tagged BTas-Y, BTas-B or BTas-D108S protein was incubated with BFV LTR DNA probes, followed by electrophoresis to resolve the protein/DNA complexes. The results showed direct interaction of BTas-B proteins with LTR probes (Fig. 5c). These interactions were lost with the addition of unlabeled LTR (20×) probes, which demonstrates the specificity of the observed interaction (Fig. 5c). In contrast, the BTas-Y protein displayed a much weaker interaction with LTR probes, compared to BTas-B and BTas-B-N108S which showed stronger binding to LTR and exhibited more potent transactivation activities (Fig. 4b). Similar results were obtained using IP probes. The purity of these GST-tagged BTas proteins used in EMSA was examined by Coomassie blue staining (Fig. 5d).

We further examined the interaction between the BTas binding domain (BD, 1–133 aa) and LTR or IP using the BFV LTR-Luc or IP-Luc reporter plasmid. The BD of BTas-Y or BTas-B was fused to the GAL4 activation domain (AD). After the co-transfection with reporter plasmids, the binding ability was evaluated by detecting the expression level of the luciferase reporter. The results showed that the fusion protein BTas-B (1–133 aa)-GAL4(AD) transactivated LTR or IP ~100 fold greater than the fusion protein BTas-Y(1–133 aa)-GAL4(AD) (Fig. 5e). Similar observations were made when experiments were performed in Cf2Th cells (Fig. 5f). Together, these findings suggest that the residue at position 108 is important for BTas binding to the BFV LTR or IP.

### Mutating N108 impairs BFV replication

**Fig. 6:**
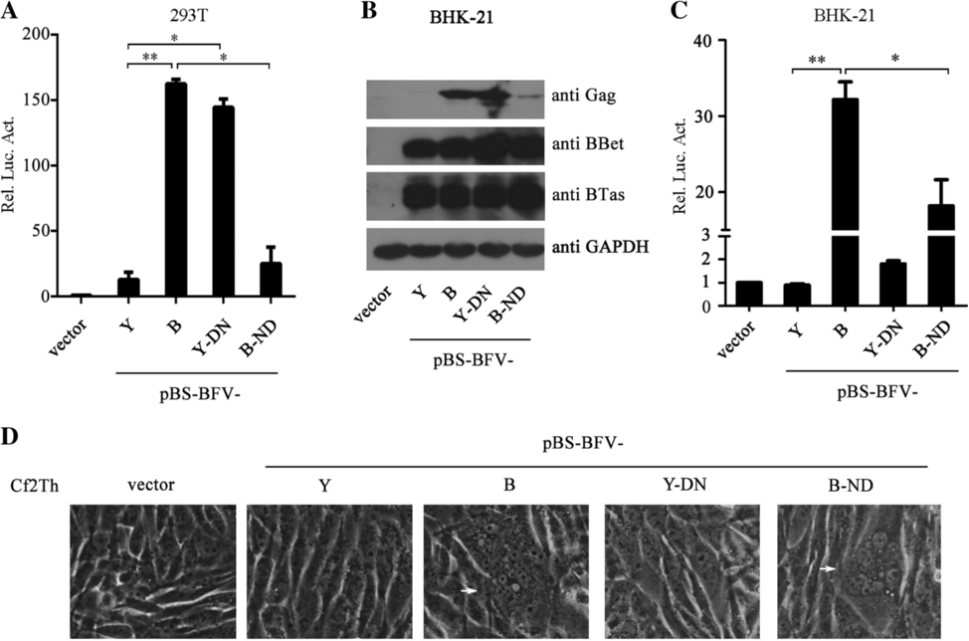
Residue N108 in BTas is important for BFV replication. **a** HEK293T cells (2.5 × 10^5^) were transfected with 1 μg pBS, pBS-BFV-Y, pBS-BFV-B, pBS-BFV-Y-D108N or pBS-BFV-B-N108D, together with pBFV-LTR-Luc and pCMV-β-gal. Forty-eight hours later, the cells were collected and analyzed using a luciferase activity assay. **b**-**c** BHK-21 cells (1.2 × 10^5^) were transfected with 1 μg pBS, pBS-BFV-Y, pBS-BFV-B, pBS-BFV-Y-D108N or pBS-BFV-B-N108D. At 48 h post-transfection, cells were harvested for Western blotting to measure viral protein expression **b**. Cells were also co-cultured with BFVL cells. Luciferase was measured 48 h post-infection **c**. **d** Cf2Th cells (1.5 × 10^5^) were transfected with 2.5 μg pBS, pBS-BFV-Y, pBS-BFV-B, pBS-BFV-Y-D108N or pBS-BFV-B-N108D. Seventy-two hours later, syncytia formation (*white arrows*) was analyzed by microscopy. All results shown are the average from three independent experiments. Error bars indicate SD. *P < 0.05 (unpaired *t* test), **P < 0.01 (unpaired *t* test)

In order to determine the importance of BTas N108 in BFV replication, two BFV mutants, pBS-BFV-Y-D108N and pBS-BFV-B-N108D, were generated, in which the amino acid residue at position 108 of BTas was substituted by N or D, respectively. First, the transactivation activity of these clones were analyzed in HEK293T cells. The results showed that the D108N mutation enhanced the transactivation activity of pBS-BFV-Y, whereas the N108D mutation impaired pBS-BFV-B transactivation (Fig. 6a). These clones were then analyzed for their replication ability in BHK-21 cells. We observed that BTas protein expression levels were similar between these clones in transfected BHK-21 cells (Fig. 6b). Gag expression closely matched the transactivation results (Fig. 6b). When the transfected BHK-21 cells were co-cultured with BFVL indicator cells, we observed that the N108D mutation impaired the replication capacity of pBS-BFV-B (Fig. 6c). However, the D108N mutation did not increase the replication of pBS-BFV-Y (Fig. 6c). In agreement with this latter observation, the pBS-BFV-B and pBS-BFV-B-N108D clones, but not pBS-BFV-Y or pBS-BFV-Y-D108N, caused syncytia in the transfected cells (Fig. 6d). Taken together, these data suggest that the residue N108 in BTas modulates the function of BTas as well as BFV replication. The results also suggest that changes in other viral genes or regions between pBS-BFV-B and pBS-BFV-Y also account for their different replication capacities.

## Discussion

BTas is a key regulator for BFV gene expression. BTas harbors a DNA binding domain and a transactivation domain, both of which are required for the transactivation function [10, 22]. In this study, the different replication abilities of two BFV3026 full-length clones were investigated. The data showed that these two viral clones differ in their BTas proteins at amino acid position 108 and that this amino acid is important for the direct interaction of BTas to LTR or IP, consequently determining the transactivation ability of BTas as well as viral replication.

Our lab isolated the BFV3026 strain from bovine peripheral blood lymphocytes in 1996 [3] and had passaged this virus in primary FBL cells. In 2002, we first cloned the *btas* gene of BFV3026 from the Hirt DNA extracted from infected FBL cells. The BTas protein encoded by this clone contains the D residue at position 108. The full-length clone pBS-BFV-Y was generated in 2003 by PCR also using DNA that was extracted from infected FBL cells. In this full-length clone, the encoded BTas protein also has D108. Since FBL cells are primary cells and must be discarded after several passages, we have been passaging the BFV3026 virus in the Cf2Th cell line since 2008. We generated 18 full-length BFV DNA clones using Hirt DNA extracted from infected Cf2Th cells in 2011, one of which was pBS-BFV-B. In addition to pBS-BFV-B, we have also sequenced the *btas* gene in five clones with low replication capacities and two clones with high replication abilities. In all of these seven clones, BTas carries the residue N at position 108. We suspected that the different residues at position 108 in BTas had resulted from the use of different cell types for replicating BFV. Thus, single genome amplification (SGA) was performed to determine the *btas* sequences from the BFV3026 viruses that were cultivated in FBL cells and had been stored at different times from 2003 to 2008. Among the 25 sequences obtained, only one BTas has D108, while the others contain N108. We also examined the *btas* genes of BFV strains from other locations and found that they bear N108 [23]. These findings indicate that N108 is a highly conserved residue of BTas, and the 108D mutation is likely a defective variant in the BFV3026 virus population. Considering D to N mutation of the amino acids at 108 site of BTas was induced by G to A mutation, it might be a reason that after long term cultivation, the viral genomes were edited by apolipoprotein B mRNA editing enzyme 3 (APOBOEC3), which could induce G to A shift.

Residue N108 is located in the DNA binding domain of the BTas protein. Results of the mutagenesis analysis support a role for N108 in the binding of BTas to viral promoters and therefore the transactivation activity of BTas. Further studies showed that N108D neither sequestered BTas in the cytoplasm or the nucleus, nor impaired the dimerization of BTas (Figs. 5 and 6). Previous studies have shown that lysines at positions 66, 109 and 110 were important for BTas binding to DNA. The K110R mutant was found to have lost binding to the BFV promoter and the transactivation ability when the 108 position was occupied by D [16]. Interestingly, the D108N substitution increased the transactivation activity of K110R by ~20-fold (data not shown). The negative charge of D or E at position 108 possibly deters the binding of BTas to the negatively charged DNA, in contrast to the positively charged patch formed by K66/109/110 that stabilizes BTas binding to DNA. We cannot rule out the possibility that N108 facilitates the adoption of an active conformation of BTas. Structural studies are expected to provide definitive answers to these possibilities.

N108 is important for the transactivation ability of BTas and protein expression of BFV, including Gag, BBet and BTas. However, the D108N mutation in the context of BFV-Y did not increase the replication ability of this replication-deficient clone (Fig. 6c and d). In addition, the BFV clones with lower replication capacities shown in Fig. 1a also harbor N108 in BTas, suggesting the existence of additional viral determinants for BFV replication. These viral replication determinants may be located in other viral genes or regions in the S3 fragment or in the S1 or S2 segments, since substitution of pBS-BFV-Y S1 and S2 also diminished the replication capacity of pBS-BFV-B (Fig. 2b). Further studies are warranted to identify these viral determinants for BFV replication.

## Conclusions

In summary, we have discovered the important role of amino acid N108 in the transactivation activity of BTas and the replication of BFV3026. This function of N108 is attributed to its modulation of the binding of BTas to viral promoter DNA.

## Abbreviations

AD, activation domain; BD, binding domain; BFV, bovine foamy virus; BTas, bovine foamy virus Tas; EMSA, Electrophoretic mobility shift assay; FBL, primary fetal bovine lung cells; IP, internal promoter; LTR, long terminal repeat promoter; pBS, pBluescript SK- vector; PFV, prototype foamy virus; SGA, single genome amplification.

## References

[1] Hooks JJ, Gibbs CJ, Chou S, Howk R, Lewis M, Gajdusek DC (1973). Isolation of a new simian foamy virus from a spider monkey brain culture. Infect Immun.

[2] Herchenroder O, Renne R, Loncar D (1994). Isolation, cloning, and sequencing of simian foamy viruses from chimpanzees (SFVcpz): high homology to human foamy virus (HFV). Virology.

[3] Liu S, Chen H, Chen J (1997). Isolation and Identification of A Bovine Spumavirus Isolate 3026. Chin J Virol.

[4] Tobaly-Tapiero J, Bittoun P, Neves M (2000). Isolation and characterization of an equine foamy virus. J Virol.

[5] Flower RLP, Wilcox GE, Cook RD, Ellis TM (1985). Detection and prevalence of serotypes of feline syncytial spumaviruses. Arch Virol.

[6] Rethwilm A, Erlwein O, Baunach G, Maurer B, ter Meulen V (1991). The transcriptional transactivator of human foamy virus maps to the bel 1 genomic region. Proc Natl Acad Sci U S A.

[7] Erlwein O, Rethwilm A (1993). BEL-1 transactivator responsive sequences in the long terminal repeat of human foamy virus. Virology.

[8] He F, Blair WS, Fukushima J, Cullen BR (1996). The human foamy virus Bel-1 transcription factor is a sequence-specific DNA binding protein. J Virol.

[9] Kang Y, Blair WS, Cullen BR (1998). Identification and functional characterization of a high-affinity Bel-1 DNA binding site located in the human foamy virus internal promoter. J Virol.

[10] Tan J, Hao P, Jia R (2010). Identification and functional characterization of BTas transactivator as a DNA-binding protein. Virology.

[11] Ma Q, Tan J, Cui X (2014). Residues R199H200 of prototype foamy virus transactivator Bel1 contribute to its binding with LTR and IP promoters but not its nuclear localization. Virology.

[12] Bodem J, Krausslich HG, Rethwilm A (2007). Acetylation of the foamy virus transactivator Tas by PCAF augments promoter-binding affinity and virus transcription. J Gen Virol.

[13] Kehl T, Tan J, Materniak M (2013). Non-Simian Foamy Viruses: Molecular Virology, Tropism and Prevalence and Zoonotic/Interspecies Transmission. Viruses.

[14] Wang J, Tan J, Guo H (2010). Bovine foamy virus transactivator BTas interacts with cellular RelB to enhance viral transcription. J Virol.

[15] Tan J, Qiao W, Xu F, Han H, Chen Q, Geng Y (2008). Dimerization of BTas is required for the transactivational activity of bovine foamy virus. Virology.

[16] Chang R, Tan J, Xu F (2011). Lysine acetylation sites in bovine foamy virus transactivator BTas are important for its DNA binding activity. Virology.

[17] Bing T, Yu H, Li Y (2014). Characterization of a full-length infectious clone of bovine foamy virus 3026. Virol Sin.

[18] Wang J, Guo HY, Jia R (2010). Preparation of BFV Gag antiserum and preliminary study on cellular distribution of BFV. Virol Sin.

[19] Bing T, Wu K, Cui X (2014). Identification and functional characterization of Bet protein as a negative regulator of BFV3026 replication. Virus Genes.

[20] Liu J, Liu S, Chen Q, Geng Y. Borf-1 protein identifi ed as a transcriptional transactivator of bovine foamy virus. Chin Sci Bull 1999;44:1017-1021.

[21] Guo HY, Liang ZB, Li Y, Tan J, Chen QM, Qiao WT (2011). A new indicator cell line established to monitor bovine foamy virus infection. Virol Sin.

[22] Liu J, Geng Y (1999). Targets for the transactivator in the long terminal repeat of bovine foamy virus. Acta scientiarum naturalium Universitatis Nankaiensis.

[23] Hechler T, Materniak M, Kehl T, Kuzmak J, Lochelt M (2012). Complete genome sequences of two novel European clade bovine foamy viruses from Germany and poland. J Virol.

